# Negative symptoms and cognitive impairment are associated with distinct motivational deficits in treatment resistant schizophrenia

**DOI:** 10.1038/s41380-023-02232-7

**Published:** 2023-08-25

**Authors:** Y. Saleh, I. Jarratt-Barnham, P. Petitet, E. Fernandez-Egea, S. G. Manohar, M. Husain

**Affiliations:** 1 Nuffield Department Clinical Neurosciences, University of Oxford, Level 6, West Wing, John Radcliffe Hospital, Oxford, OX3 9DU UK; 2 Department of Psychiatry, University of Cambridge, Herchel Smith Building for Brain & Mind Sciences, Forvie Site, Robinson Way, Cambridge, CB2 0SZ UK; 3 Cambridge Psychosis Centre, Cambridgeshire and Peterborough NHS Foundation Trust, Cambridge, UK; 4 Centre de Recherche en Neurosciences de Lyon, Equipe Trajectories, Inserm UMR-S 1028, CNRS UMR 5292, Universite Lyon 1, Bron, France; 5 Department of Experimental Psychology, University of Oxford, Oxford, UK

**Keywords:** Biological sciences, Schizophrenia

## Abstract

**Background:**

Motivational deficits are a central feature of the negative syndrome in schizophrenia. They have consistently been associated with reduced willingness to expend physical effort in return for monetary rewards on effort based decision making (EBDM) paradigms. Nevertheless, the mechanisms underlying such altered performance are not well characterised, and it remains unclear if they are driven purely by negative symptoms, or also in part by cognitive impairment, antipsychotic treatment or even positive symptoms. Here we investigated the impact of all these factors using a paradigm that has not previously been used to measure EBDM in schizophrenia.

**Methods:**

Forty treatment resistant schizophrenia (TRS) patients on clozapine and matched controls *(N* = 80) completed a well validated EBDM task which offers monetary rewards in return for physical effort. Choice and reaction time data was analysed using logistic regressions, as well as Bayesian hierarchical drift diffusion modelling (HDDM). Behavioural parameters were compared between groups and their association with negative symptoms, cognitive function and serum clozapine levels were assessed.

**Results:**

Overall, TRS patients accepted significantly less offers than controls during effort-based decision making, suggesting they were less motivated. They demonstrated reduced sensitivity to increasing rewards, but surprisingly were also less averse to increasing effort. Despite a positive correlation between negative symptoms and cognitive function in TRS, reward sensitivity was associated only with cognitive performance. In contrast, reduced effort aversion correlated with negative symptom severity. Clozapine levels and positive symptoms were not associated with either behavioural parameter.

**Conclusion:**

Motivational deficits in TRS are characterised by both diminished reward sensitivity and reduced effort aversion during EBDM. Cognitive dysfunction and negative symptom severity account for distinct aspects of these behavioural changes, despite positive associations between themselves. Overall, these findings demonstrate that negative symptoms and cognitive impairment have significant independent contributions to EBDM in TRS, thereby opening the possibility of individualised treatment targeting these mechanisms to improve motivation.

## Introduction

Negative symptoms carry a high burden of disease in schizophrenia [1–5], where they occur in up to 60% of patients [6]. In treatment resistant schizophrenia (TRS), which is characterised by non-response to two or more antipsychotics, patients often suffer with an increase in both frequency and severity of negative symptoms [7]. While other symptoms are also prominent in TRS, such as positive symptoms (i.e. hallucinations/delusions) [7], recent works suggests that disease severity in this sub-group may be mediated by negative symptom severity [8]. Despite their significant impact, there are no effective interventions for negative symptoms, and they are typically non-responsive to antipsychotic therapy [9]. Clearly, they represent an unmet therapeutic need [10], yet their underlying mechanisms remain poorly understood.

Motivational deficits are a central feature of the negative syndrome [11–13], and represent a critical treatment target in schizophrenia [11]. They include decreased goal directed behaviour (avolition), reduced socialisation (asociality), and a lack of pleasure when anticipating or engaging in activities (anhedonia) [10]. Other symptoms include minimal speech production (alogia) and blunted affect [10]. Factor analyses of questionnaire reports have often supported a two-factor structure for negative symptoms [13–17]. These consist of a motivation and pleasure factor (MAP) which includes avolition, anhedonia, and asociality; and a blunted self expression factor (EXP), which consists of alogia and blunted affect [13–17]. While these analyses are insightful, they do not clarify exactly how motivational deficits or blunted expression contribute towards pathological behaviour. A deeper understanding of these symptoms is needed to shed light on their underlying mechanisms and lay foundations for future treatments.

One framework used to conceptualise motivated behaviour in schizophrenia is that of effort based decision making for rewards (EBDM) [18, 19]. Tasks that probe people’s willingness to expend effort for reward, often inspired by pre-clinical studies, investigate the behavioural mechanisms of motivational deficits by probing the different phases of decision making [18]. One example is the process of option selection, where participants are asked to weigh up the benefits (e.g., potential reward) and costs (e.g., effort required to obtain that reward) of a decision prior to initiating goal-directed action [18, 20, 21]. A commonly used option selection paradigm in Schizophrenia is the ‘Effort Expenditure for Rewards Task’ (EEfRT) [22–25], where people are asked to choose between a low reward–low effort option or a high reward–high effort one [22]. Compared to healthy controls, patients with schizophrenia select significantly fewer high effort offers when the reward levels are at their highest [23–28]. Some studies have found that this behavioural pattern correlates with negative symptom severity [26] or specifically amotivation [24, 25, 29, 30]. Nevertheless, several important questions remain unanswered.

First, it is not entirely clear exactly why patients with schizophrenia performing the EEfRT task accept fewer offers than controls when rewards are at their *highest* levels. Patients may misrepresent value because they are less responsive, or insensitive, to reward; more averse, or hypersensitive, to effort; or intrinsically unmotivated. It remains unclear which of these possibilities might best account for the behaviour observed. One critique of the EEfRT task, which relates to these issues, is that it does not simultaneously vary the amount of reward and effort within its experimental structure, often using five reward levels but only two effort levels [25]. Paradigms with more symmetrical task structures might help disentangle the precise contributions of reward and effort through the extraction of behavioural parameters such as reward sensitivity, effort sensitivity and intrinsic motivation [31]. These measures, derived from EBDM data, have been used to characterise clinical apathy in Parkinson’s [32] and cerebrovascular disease [33, 34], adding valuable insights into their underlying mechanisms.

Second, while patients with schizophrenia frequently suffer from cognitive deficits [35–37], the relationship between cognition and negative symptoms is unclear [38]. Analyses of questionnaire data demonstrate positive associations between cognitive dysfunction and negative symptom severity [39, 40], although these have been described as modest [40]. Further, like negative symptoms, cognitive deficits in schizophrenia are also associated with impaired reward based decision making [24, 30, 41, 42]. So what is the relationship between negative symptoms and cognitive impairment?

One view is that they may be independent constructs that share a similar aetiology [38, 43]. Moreover, Robison et al. propose that synergistic interactions between cognitive deficits and negative symptoms may drive motivational impairments in schizophrenia [43]. Recent re-analyses of EBDM data in schizophrenia by Cooper et al. showed that cognitive impairment and negative symptom severity might both alter motivated behaviour through independent, yet complementary mechanisms [30]. Specifically, cognitive dysfunction was associated with the inability to incorporate information about reward. On the other hand, negative symptoms related to motivation were associated with increased effort aversion [30]. However, there were several limitations with the findings. First, the behavioural effect associated with cognitively impaired patients was unclear. Did they fail to process information about rewards in isolation or were they unable to *integrate* reward and effort cues? This was difficult to dissociate as the task did not systematically vary these two variables [30]. Second, the correlation between amotivation and effort aversion was limited to a subgroup accounting for ~50% of patients. Moreover, this group actually had higher cognitive scores, and were more able to utilise all task information systematically according to computational modelling analyses. The way that effort was processed in the other 50% of the patients, however, remains unclear.

TRS patients may represent a valuable cohort when attempting to clarify some of these questions. For example, they suffer with more severe negative symptoms and cognitive deficits compared to their treatment responsive counterparts [7, 44]. So the hypothesis that cognitive impairment and negative symptoms are associated with diverging behavioural phenotypes can be directly tested in this patient group. Interestingly, while they account for up to 30% of all patients with schizophrenia [45, 46], few EBDM studies to our knowledge have attempted to characterise the behavioural deficits in TRS. So it is not clear if the behavioural deficits described in the literature previously using the EEfRT task [25–28, 30] are also present in TRS. Do they also accept significantly less offers at high rewards/high effort when performing an option selection task [23, 26, 30, 47, 48]? If so, is this behaviour accounted for by a specific deficit in processing reward, effort, or a combination of the two? Finally, what are the individual contributions of negative symptoms and cognitive impairment towards these behaviours?

Clarifying these questions will shed valuable light on the behavioural mechanisms of not just TRS but patients with schizophrenia as a whole. For example, while some groups suggest that TRS patients are categorically distinct entity [49], few EBDM investigations have attempted to clarify whether they have a unique behavioural phenotype. A recent study by Horne and colleagues was not able to demonstrate statistically significant differences between treatment responsive and resistant patients during a reinforcement learning paradigm, despite both groups performing worse than controls [44]. One critique of that work was that the administered task was technically difficult to perform, and possibly unable to unpick subtle differences within group differences. Alternatively, it is possible that the behavioural deficits in TRS overlap with those in treatment responsive patients.

To answer these questions we conducted an EBDM study in a group of 40 TRS patients on clozapine treatment which requires therapeutic monitoring through plasma concentration measurements [50]. This involved conducting an EBDM task which parametrically modulates reward on offer and effort required, and has previously been used to assess motivation in neurological disorders [31–34].

We had two apriori hypotheses:

1. TRS patients accept significantly less offers during option selection, specifically when reward and effort levels are highest. This is driven by individual deficits in both reward and effort sensitivities.
2. Negative symptom severity and cognitive impairment, while positive correlated, are associated with distinct behavioural deficits, as suggested by recent reanalyses of EEfRT data [30]. Specifically, negative symptoms are associated with altered effort sensitivity, while cognitive impairment is associated with reward insensitivity.

We used a combination of analytic approaches, including a well validated computational modelling approach known as drift diffusion modelling (DDM), which integrates both choice behaviour and reaction time metrics. This has been recently used to characterise apathetic behaviour in cerebrovascular small vessel disease (SVD), as well successfully correlate the behavioural deficits of apathy in SVD to its neural correlates [34].

## Methods

### Participants

Forty outpatients with TRS were recruited from a clozapine clinic in the Cambridgeshire and Peterborough NHS foundation Trust. Disease severity in this patient group is significantly mediated by negative symptoms [8]. This is a sample size consistent with seminal papers investigating effort-based decision making in schizophrenia [23, 24, 26, 29 and Supp. Table 1]. Treatment resistance was defined as the persistence of psychotic symptoms after treatment with two different antipsychotics at the appropriate dose for a duration of at least four weeks. Participants were clinically stable, with no medication dose changes in the last eight weeks. They were recruited during routine monthly blood-tests between August 2018 and March 2019. Additionally, forty healthy-age and gender-matched controls were recruited over the same time course. Inclusion and exclusion criteria can be found in the supplementary methods.

### Cognitive and questionnaire measures

Negative symptoms in patients were measured using the clinician administered brief negative symptom scale (BNSS) [51]. Factors derived from a previous investigation of 146 patients with schizophrenia were used as the MAP (motivation and pleasure which includes avolition, anhedonia, and asociality) and EXP (self expression which includes alogia and blunted affect) negative symptom factors for our patient group [17]. Patients showed higher mean MAP scores, although paired t-tests showed this was not significant [*t*(39) = 1.86, *p* = 0.09]. Similarly, the standard deviations of both factors across the groups were similar (respectively, 7.39 and 7.29).

Cognitive function was quantified using both the Addenbrooke’s Cognitive Examination (ACE-III) [52, 53] and the composite score of the brief assessment for cognition in schizophrenia (BACS) [54]. Depression was measured using the Calgary depression scale (CDS) [55], and positive symptoms using the positive subscale (P1–P7) of the positive and negative syndrome scale for schizophrenia (PANSS) [56]. Clozapine dose, plasma clozapine levels, and Olanzapine dose equivalents for other antipsychotic medications were additionally recorded.

### Experimental design

**Fig. 1:**
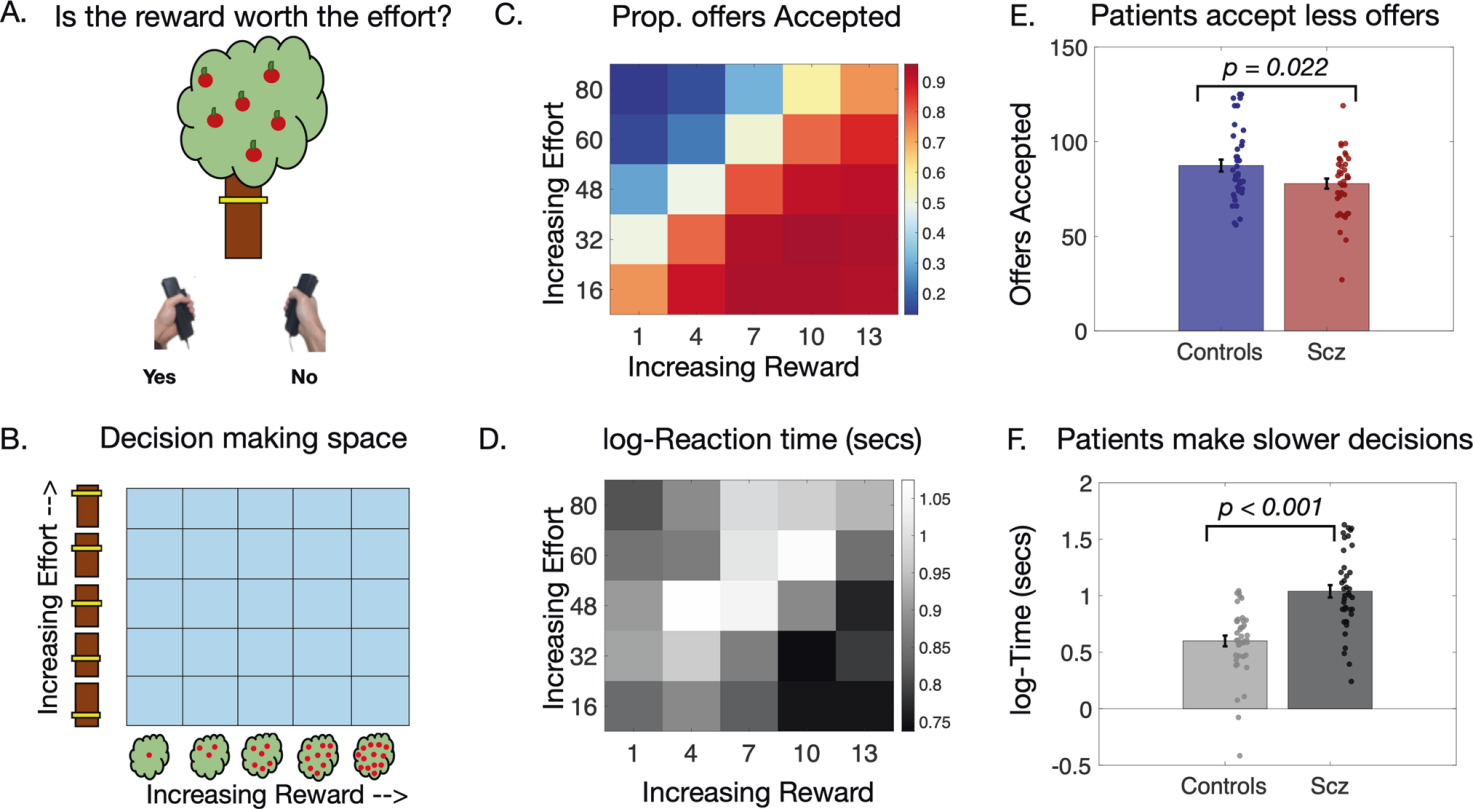
Effort based decision making task and groupwise results. **A** Participants completed a computer task which offered monetary rewards (virtual apples) in return for physical effort (height of the yellow bar). **B** Five different levels of reward and effort were pseudorandomised throughout the paradigm (permutations mapped on a two-dimensional decision space of reward and effort 5 × 5 grid). **C, D** Acceptance rates and reactions times within each segment of the decision space for all 80 participants. **C** More offers were accepted at as the rewards increased (heatmap becomes more red from left to right). Inversely more offers were rejected at high effort (heatmap more blue from bottom to top). **D** Decisions were slowest (lighter colours) when the levels of reward and effort were at their intermediate levels. Otherwise, offers were accepted and rejected more quickly (darker colours) at the extreme ends of reward and effort. **E** Patients accepted fewer offers overall compared to controls. **F** They were also significantly slower across all decisions.

Participants completed an ‘Apple Gathering’ task (Fig. 1) previously used to investigate motivated behaviour in patients with Parkinson’s and small vessel cerebrovascular disease [31, 32, 34]. This was designed in psychtoolbox (psychtoolbox.org) using MATLAB and administered on a mounted laptop [32, 34]. Participants were offered monetary reward in return for physical effort which involved squeezing a hand-held force dynamometer (SS25LA, BIOPAC Systems) [34]. Reward magnitude was specified by the number virtual apples on a tree (1, 4, 7, 10, or 13), and effort by the height of a yellow bar on the tree trunk (Fig. 1A) [34]. The force required was derived from each participant’s own maximum voluntary contraction (10, 36, 48, 64, or 80%), computed in a ‘calibration phase’. During the experiment, offers could be accepted/rejected by pressing the left/right buttons on the laptop keyboard. Overall, this made it possible to assess their willingness to work for different levels of reward and effort, across a reward × effort decision making space (Fig. 1B). Participants were not required to squeeze for every single accepted choice. Instead, ten choices were randomly selected at the end of the experiment and if they had chosen ’Yes’, they would have to expend the appropriate amount of effort to secure the trial specific rewards. Based on the number of apples collected on these trials, they were told that they would receive a cash prize. In practice, all participants were rewarded with a fixed amount based on an hourly rate (~10 GBP/h).

With five levels of reward and effort, 25 possible offer types were available. Each offer was sampled five times, giving a total of 125 decisions divided into five blocks of 25 trials. Trial order was pseudo-randomised, ensuring all participants were presented choices in the same order. Before the experiment, each participant practiced squeezing the handheld device at each effort level and completed three practice decisions. The extracted behavioural parameters were choice (i.e. accept or reject) and reaction time.

### Analyses

#### Clinical measures and factor analyses

Pearson correlations were used to establish within group associations between questionnaire measures, including the factor weighted questionnaire scores. Clinical measures were subsequently used as predictors of the behavioural parameters outlined below.

#### Between group behavioural comparisons

Groupwise comparisons of accepted offers and log-transformed reaction times were conducted using two-sample t-tests. Logistic regressions with mixed effects were used for analysis of behavioural mechanisms. Two initial models were run to determine the behavioural differences between patients and controls (see supp. tables 2–1 and 2-2). These included all interactions between reward and effort and the fixed effects of patient group. Model fits were assessed using the Akaike information criterion. Statistical significance was inferred when *P* values were <0.05. Behavioural parameters of interest were:

1. Intrinsic motivation—general tendency to accept offers, represented by $\kappa$.
2. Reward sensitivity—responsiveness to increasing reward, represented by $\alpha$.
3. Effort sensitivity—aversion to increasing effort, represented by ${{{{{\rm{\beta }}}}}}$.
4. Reward:effort interaction term—ability to integrate reward and effort related information, represented by ${{{{{\rm{\gamma }}}}}}$.

These were used to model behaviour across the whole group by computing subjective value (sv) and choice probability (*p)* using the following equations:

1 $${sv}=\kappa +\alpha {{{{{\rm{Reward}}}}}}+{{{{{\rm{\beta }}}}}}{{{{{\rm{Effort}}}}}}+{{{{{\rm{\gamma }}}}}}{{{{{\rm{Reward}}}}}}:{{{{{\rm{Effort}}}}}}$$

2 $$p=1/1+{e}^{-{sv}}$$

Within-group logistic regressions were conducted to assess the independent effects of cognitive function, total negative symptoms, and both negative symptom subcomponents. Family-wise error rate correction was conducted for four multiple comparisons using the Bonferroni method [57]. Multiple regressions were conducted between behavioural parameters and negative symptom subcomponents, positive symptoms, cognitive function, depression and clozapine levels. Error bars on all plots were represented by the standard error of the mean.

### Hierarchical drift-diffusion modelling of effort-based decision making

**Fig. 2:**
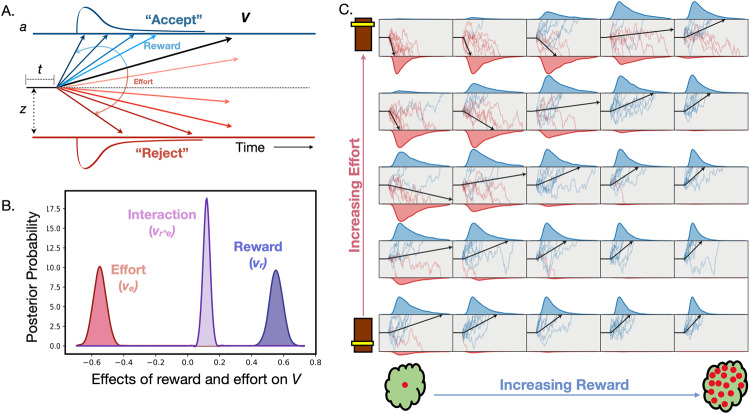
Drift diffusion model (DDM) of effort-based decision making for reward. **A** Decision making is modelled as a noisy process of evidence accumulation up to a decision boundary, *a*, occurring at a drift rate, *V*, from a starting point termed the bias, *z*. Time spent in non-decision making processes is encapsulated by a non-decision term, *t*. **B** Reward and effort both significantly altered drift rate. **C** A simulation of the influence of reward (x-axis) and effort (y-axis) on drift rate (black line) within our decision space. There is an incremental rise in drift rate with increasing reward (left to right), and fall in drift rate with increasing effort (bottom to top).

A Bayesian drift diffusion model (DDM) was fit to both the reaction time and choice data [58] (http://ski.clps.brown.edu/hddm_docs/; version 0.8.0; Python 3.6). This model frames participants’ decision making process as a noisy accumulation of evidence towards one of two decision boundaries, beyond which a decision is made (i.e. accept or reject, Fig. 2A). The model can be encapsulated in four broad parameters: i) *bias*, *z*, which determines the a priori starting point of evidence accumulation; ii) *threshold*, *a*, which represents the distance between the two decision boundaries; iii) *non decision time*, *t*, which accounts for biological processes not actively contributing to the decision making process (e.g. sensory perception, motor execution); iv) *drift rate*, *v*, which speaks to the rate of evidence accumulation. The individual effects of reward and effort on the drift rate parameter were also included. Groupwise comparison of parameters was assessed using Bayesian inference, reported using posterior probabilities (*P*_P|D_) of hypotheses of interest. A posterior probability of 0.95 or more was deemed significant. A full description of the model specifications and evaluation (Supp. Figs. 1–2 and supp. table 3) can be found in the supplementary methods section. Exploratory multiple regressions were repeated between each DDM parameter and: negative symptoms, cognitive function, depression, and clozapine level.

## Results

### Reduced responsiveness to high rewards, decreased aversion to high effort in TRS

**Fig. 3:**
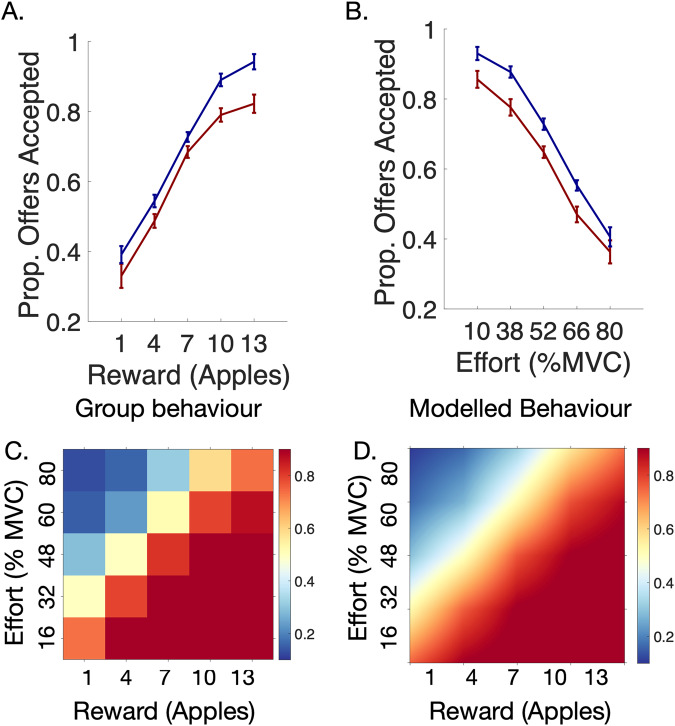
Performance and modelling of data. **A,** **B** Patients accepted fewer offers compared to healthy participants. This was especially the case at high reward and low effort. Controls were more sensitive to increasing effort than patients. **C** Raw data for all 80 participants across the entire decision making space. **D** Modelled behaviour for all participants.

Patients and controls did not significantly differ in age nor gender. Overall, TRS patients were significantly more cognitively impaired as indexed by ACE-III scores (*t*(77) = 5.71, *p* < 0.001, Table 1). They accepted significantly fewer offers and had prolonged reaction times in comparison to controls (respectively, *t*(78) = −2.32, *p* = 0.023 and *t*(78) = + 6.06, *p* < 0.001; Fig. 1E, F). Our chosen logistic regression model (Model 1, Supp. Table 2–1) demonstrated that patients accepted significantly fewer offers overall [*F*(1,9977) = 7.66, *P* = 0.0056]. Additionally, there was a two way interaction between Schizophrenia*Reward and Schizophrenia*Effort (respectively, [*F*(1,9977) = 4.94, *P* = 0.026] and [*F*(1,9977) = 3.96, *P* = 0.046]. Specifically, TRS patients accepted significantly fewer offers than controls at the *highest* levels of reward (Fig. 3A). On the other hand, as effort progressively increased, they rejected proportionally fewer offers when compared to healthy controls (Fig. 3B). There was no three way interaction between patient group, reward and effort [*F*(1,9977) = 0.27, *P* = 0.60]. Adding cognitive scores as a covariate in this model abolished the groupwise effects. Notably, only reward sensitivity decreased with greater cognitive impairment [*F*(1,9848) = 9.49, *P* = 0.0021], but not effort sensitivity [*F*(1,9848) = 1.3, *P* = 0.2]. In summary, TRS patients were less sensitive to high rewards, and less averse to high effort. While cognitive impairment was significantly associated with diminished sensitivity to reward, it was not associated with blunted aversion to effort.

**Table 1:** Demographics table.

	Control (*n* = 40)	Schizophrenia (*n* = 40)	*p* value
Age	42.78 (12.72)	46.35 (10.12)	0.17
Gender (M/F)	39/1	36/4	0.17
Illness duration	—	24.65 (8.53)	—
ACE-III^a^	95.5 (5.94)	84.6 (10.45)	*<0.001
BACS	—	35.83 (13.44)	—
BNSS total	—	20.9 (17.25)	—
BNSS EXP	—	7.6 (7.29)	—
BNSS MAP	—	8.93 (7.39)	—
CDS	—	3.15 (3.14)	—
PANSS Total	—	56.83 (14.21)	—
PANSS (Positive)	—	12.52 (4.2)	—
Serum clozapine^a^	—	0.36 (0.17)	—

Statistics are presented as mean (SD).

*implies statistical significance.

Chi-squared test used to compare gender across groups.

Paired t-tests used for remaining measures.

^a^Data missing for one participant.

### Negative symptom severity positively associated with cognitive impairment

Pearson correlations between clinical measures within the TRS patient group are provided in Supp. Fig. 3. Total negative symptom severity (BNSS total) was significantly negatively associated with the BACS (composite score), but not the ACE-III total score (respectively, *r*(38) = −0.38, *p* = 0.02 and *r*(38) = −0.28, *p* = 0.079). The factor weighted MAP and EXP were correlated with one another *r*(38) = 0.77, *p* < 0.0001). Notably, only the EXP but not the MAP component was significantly negatively associated with both the BACS and ACE-III cognitive scores (respectively, *r*(38) = −0.43, *p* = 0.009 and *r*(38) = −0.32, *p* = 0.045). Serum clozapine was not associated with any clinical measures. Together, these findings show that in TRS, the greater the total negative symptom severity, the worse the cognitive function. Additionally, this seems to be driven by blunted expression (EXP factor), rather than the motivation and pleasure (MAP) component of negative symptoms.

### Relationship of behavioural parameters to cognition and negative symptoms

**Fig. 4:**
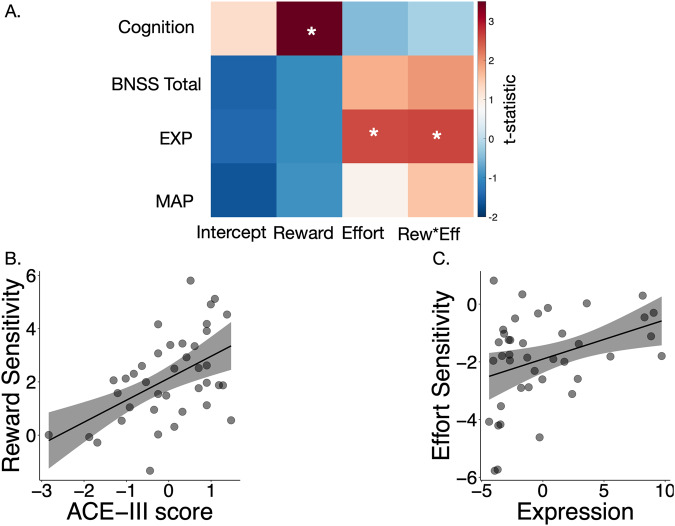
Relationship of behavioural parameters to clinical measures. **A** T-statistic map showing associations between the four behavioural parameters and clinical measures of interest (respectively, along the *x* and *y* axes). **B** Robust regression demonstrating the positive association between cognitive function and reward sensitivity. **C** Association between blunted self-expression score and effort sensitivity.

We focussed on the ACE-III, instead of the BACS as the main cognitive measure of interest as it was more sensitive to behavioural measures (See supp. results section: ‘Cognitive variables’). Within group analyses showed that *reward sensitivity* was positively associated with the ACE-III score (Fig. 4A, B) after correcting for four multiple comparisons [*F* (1,4852) = 13.39, *P* = 0.0008]. Post-Hoc analysis revealed that all but the fluency sub-domains were significantly positively associated with the reward parameter (see Supp. Table 4). Reward sensitivity was also positively associated with the BACS questionnaire, although this correlation was weaker [*F*(1,4852) = 4.481, *P* = 0.035] and did not survive multiple comparison in multiple regression analyses.

On the other hand, *effort sensitivity* was negatively associated with the EXP but not the MAP component (Fig. 4A) of the negative syndrome (respectively, [*F* (1,4977) = 6.6, *P* = 0.036] and [*F* (1,4977) = 0.78, *P* = 0.38]). Thus, patients with little or no sensitivity to effort (blunted effort sensitivity) had the highest EXP scores (Fig. 4C). There was also a three way interaction between reward*effort*EXP [*F* (1,4977) = 6.76, *P* = 0.036, after multiple comparison]. Neither parameter was associated with total negative symptom score as measured by the BNSS.

Multiple regressions were conducted between behavioural parameters and the following six variables: Cognitive function (ACE-III), negative symptom factors (EXP and MAP), depression (CDS), positive symptoms (PANS positive subscale), and clozapine level (see Supp. Table 5). ACE-III score was significantly associated with reward sensitivity (*β* = 0.87, *p* = 0.014 after multiple comparison). Similarly, the EXP negative symptom factor was negatively associated with effort sensitivity (*β* = 0.28, *p* = 0.036 after multiple comparison). These findings were replicated when additionally including olanzapine equivalents for other anti-psychotic medications. There were no significant associations between behavioural parameters and other clinical measures after correcting for multiple comparison.

**Fig. 5:**
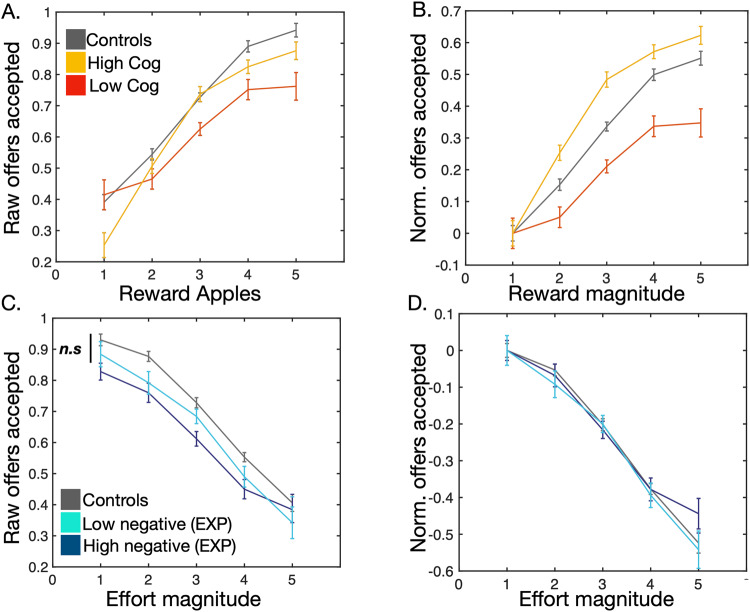
Subgroup analyses of patients with cognitive impairment or high negative symptom burden. Comparison of median split TRS subgroups to controls using ACE-III (**A**, **B**) and EXP factor scores (**C**, **D**). Two plot variations are shown, one using raw offers accepted (**A**, **C**) and another using values normalised by baseline acceptance rate (**B**, **D**). **A,** **B** Cognitively impaired patients (red) accepted less offers and were less reward sensitive compared to controls (grey). Cognitively intact patients (yellow) were not significantly different to controls. Patients with high negative symptom burden (dark blue) accepted less offers than controls (**C**) and were less effort averse at the highest effort level (best seen in normalised figure, **D**).

Sub-group analysis was then conducted to assess if our two clinical variables (Cognitive function and EXP) could be used to retrieve the groupwise differences between TRS patients and controls. Four sub-groups were generated using median splits of ACE-III and EXP score. This gave rise to cognitively impaired; cognitively intact, high blunted expression; and low blunted expression sub-groups. Each group was compared to controls, and p values corrected for the four multiple comparisons. We did not compare the two subgroups to one another as this analysis would have been accounted for more accurately using the continuous measure within group analyses above. Cognitively impaired TRS patients, accepted fewer offers overall and were less reward sensitive than controls after correcting for four multiple comparisons (respectively, [*F* (1,7356) = 12.76, *P* = 0.0016] and [*F* (1,7356) = 11.3, *P* = 0.0032], Fig. 5A, B) . Cognitively intact TRS patients did not differ from controls across behavioural parameters (respectively, [*F* (1,7613) = 0.755, *P* = 0.76] and [*F* (1,7613) = 0.006, *P* = 1], Fig. 5A, B). On the other hand, TRS patients with high EXP scores accepted fewer offers overall and showed blunted effort sensitivity compared to controls (respectively, [*F* (1,7481) = 8.1, *P* = 0.018] and [*F* (1,7481) = 7.15, *P* = 0.0028], Fig. 5C, D). TRS patients with low EXP scores did not differ from controls across parameters.

Taken together, these analyses suggest that two distinct mechanisms characterise TRS patients from controls. These are reward insensitivity and blunted responsiveness to effort. Despite positive associations between cognitive dysfunction and negative symptoms within patients, each construct was distinctly associated with one of these behavioural deficits suggesting different underlying mechanisms.

### Drift diffusion modelling

**Fig. 6:**
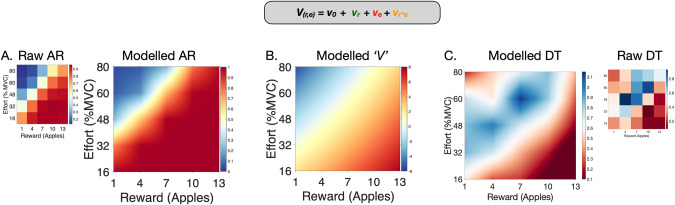
Prediction of behavioural changes using DDM parameters. Model estimates of drift rate accurately predict acceptance rate (AR) (**A**) and decision times (DT) (**C**) when compared to the raw data. Predictions for AR and DT are both derived from the actual drift rate *V*_*r,e*_ (**B**) which reflects increasing and decreasing drift rate values with reward and effort respectively.

**Fig. 7:**
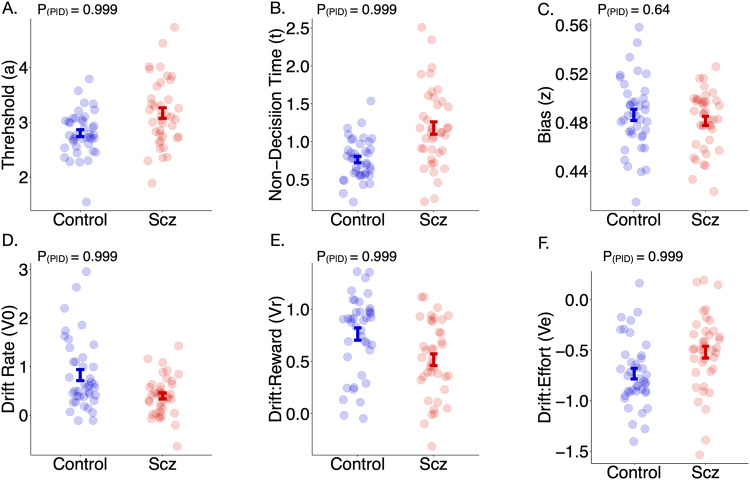
Drift diffusion parameter estimate comparison between patients and controls. Drift diffusion modelling parameters, split by group and posterior distributions compared using Bayesian hypothesis testing. Visualisations are of mean + SEM. Significant probability of differences between groups is set at 0.95. Patients with schizophrenia had significantly higher thresholds (**A**) and non decision times (**B**) but were not more biased towards rejecting offers (**C**). They also had lower overall drift rates (**D**) and showed impaired modulation of drift rate in response to both reward (**E**) and effort (**F**).

A hierarchical DDM was fitted to the data using Bayesian statistical methods. Probability distributions were generated, providing a measure of uncertainty for each parameter estimate (Fig. 2A–C). Both reward and effort significantly affected drift rate to decision threshold such that drift rate rose with increasing reward and fell with increasing effort (Fig. 2B). Parameters were used to accurately model acceptance rate (AR) and Decision time (DT) (Fig. 6). Groupwise comparison between parameters was conducted using Bayesian hypothesis testing (Fig. 7). Overall, patients with schizophrenia had significantly larger threshold (*a*) and non-decision time (*t*) values (*P*_P|D_ ≈ 1 for both parameters). Conversely, they had lower overall baseline drift rates (*P*_P|D_ ≈ 1). Namely, they were less likely to accept more offers as reward increased (reduced v*reward interaction, *P*_P|D_ ≈ 1) and less likely to reject offers as effort increased (v*Effort interaction was closer to 0 in relation to controls, *P*_P|D_ ≈ 1). There was no difference between controls and patients with schizophrenia in the v*Reward*Effort interaction (*P*_P|D_ ≈ 0.13), or bias (*z*) (*P*_P|D_ ≈ 0.74). Multiple regressions between DDM parameters and clinical measures revealed similar findings (See Supp. Table 6). Specifically, ACE-III significantly positively associated with the reward parameter (*V*_*r*_*)* whereas blunted expression (EXP) was negatively associated with the effort sensitivity parameter (*V*_*e*_), although the latter association was not significant after six multiple comparisons (*p* = 0.09).

We conducted a post-hoc analysis to investigate if any behavioural parameters were associated with MAP, and not EXP. This showed a significant difference between the overall drift rate (*V*_0_), and MAP but not EXP (respectively, [*F*(1,38) = 4.3, *p* = 0.044] and [*F*(1,38) = 1.5, *p* = 0.22]), however this association was abolished when including other clinical variables and correcting for multiple comparison.

## Discussion

The results presented demonstrate that patients with treatment resistant schizophrenia were less motivated than healthy controls when performing an EBDM task. This behaviour was characterised by reduced incentivisation to reward and blunted aversion to effort (Fig. 2A, B). Drift diffusion modelling of the choice and reaction time data (DDM) confirmed these findings while revealing the following groupwise differences (Fig. 7). Patients needed to accumulate more evidence than healthy volunteers prior to initiating rewarded effortful decisions (increased *a*), and their rate of evidence accumulation was slower (reduced *v*_*0*_). This parameter was less responsive to changes in both reward and effort (reduced *v*_*r*_ and less negative *v*_*e*_, respectively). Despite this, they did not show an indiscriminate propensity, or bias (*z*), towards rejecting offers. Within group analyses of clinical measures and behavioural parameters highlighted two further findings. First, cognitive impairment was the single best predictor of reward insensitivity (Fig. 4A, B). This was supported by both choice behaviour analysis as well as DDM, which incorporated reaction time data. Second, negative symptom severity as defined by the EXP factor best explained blunted responsiveness to effort (Fig. 4A, C). While this effect was demonstrated in choice behaviour, it was not present in the DDM analysis.

Several studies have shown a reduced responsiveness to *high* values of reward across the schizophrenic disease spectrum [23, 25, 26, 59]. This includes in first episode psychosis [59], established schizophrenia, or schizoaffective disorder [23, 25, 26]. Here we confirmed these findings in patients with TRS, demonstrating that there are common behavioural deficits across the schizophrenic spectrum. Additionally, we showed that TRS is characterised by blunted responsiveness to high effort, with patients accepting significantly less offers than controls at low effort but not at high effort (Fig. 2B). A plausible interpretation of this is that they display inefficient effort allocation during EBDM. This finding is less commonly reported [59, 60] and may be a unique characteristic of TRS patients. On the other hand, it is also possible that our task design which co-varies reward and effort across five levels is better able to detect this more subtle effect. Some EBDM paradigms in schizophrenia commonly only vary five reward values across only two effort levels (easy vs hard) [23, 25] and recent work has highlighted this as a possible limitation in understanding how reward and effort processing are individually affected in schizophrenia [30].

Within our patient group we demonstrated that reward sensitivity was positively associated with cognitive impairment, while effort insensitivity correlated with negative symptom severity (as measured by blunted self-expression). Notably, these two constructs were significantly associated with one another on questionnaire correlation (Supp. Fig. 3). So despite their association, they were characterised by unique behavioural deficits, suggesting that they may respond to different treatment strategies.

A similar divergence in behaviour was recently characterised by Cooper and colleagues [30], who reanalysed EEfRT task data from two previously published studies [25, 27]. They were able to demonstrate that in 153 patients with schizophrenia pooled across two studies individuals unable to incorporate reward related information during EBDM were characterised by cognitive impairment, even after accounting for negative symptoms. On the other hand, sub-group analysis demonstrated that some patients with motivational deficits were more effort averse [30]. Our findings diverge from that study in that while they demonstrated an *increase* in effort aversion with increasing negative symptoms, we demonstrated the opposite effect. Further, we showed that EXP, and not MAP, was associated with these changes.

There are several possible explanations for the differences in our studies. First, Cooper et al. did not investigate associations between behavioural parameters and the EXP factor [30]. Rather, they investigated the MAP dimensions while accounting for total negative symptom scores [30]. It is not clear if the EXP factor would have demonstrated an association in their study. Further, the association between amotivation and increased effort sensitivity was demonstrated in only half of their patients [30]. Thirdly, as they used pooled questionnaire data from previous studies, negative symptoms were measured using three different questionnaires. Two of these, the scale for the assessment of negative symptoms [61] and the negative component of the PANSS [56], were developed more than thirty years ago and do not reflect the current understanding of the negative syndrome [10, 62]. Nevertheless, the authors were also able to demonstrate this association in a sub-group of their patients using a ‘next-generation’ negative symptom questionnaire known as the Clinical Assessment Interview for Negative Symptoms [30]. A recent comparison between this and the BNSS questionnaire, which we used, showed that while both demonstrated good psychometric properties, there was low to moderate convergence between the two questionnaires on motivation and pleasure questions [63]. It is possible therefore that the BNSS MAP factor was not able to detect this effect in our patient group.

More generally, the association between negative symptom severity and increased effort sensitivity is actually quite variable [22–25,29, 52, 53, 57]. Indeed, several studies have failed to demonstrate this association at all [23, 24, 60] whereas others have only done so using categorical, not correlational approaches [26, 59]. These inconsistencies are possibly due to variations in task design, analytic approaches and questionnaires used to measure negative symptoms. The association between inefficient effort allocation and negative symptoms might also be explained by these technical differences. Nevertheless, this finding has also been previously described by two studies [59, 60]. In 48 patients with Schizophrenia, McCarthy and colleagues demonstrated that blunted self expression, indexed by alogia, was associated with inappropriate allocation of high effort regardless of reward value [60]. More recently, Chang et al. showed that this behavioural pattern was associated with increasing negative symptom severity, indexed by motivation and pleasure [59].

Taken together, our findings support the possibility that negative symptoms are not exclusively associated with increased effort aversion, but also with inefficient effort expenditure. Additionally, they suggest that the EXP component of the negative syndrome may also play an important role in characterising behavioural deficits in patients with schizophrenia. Nevertheless, the inconsistency across studies is important to note and steps towards resolving these will play an important role in clarifying exactly what kind of behavioural deficits characterise negative symptoms in schizophrenia. Standardised questionnaires, task designs, and analytic approaches would all be useful to reduce variation between studies and improve interpretation.

An important consideration is whether our findings are applicable to TRS patients only or whether they also have implications for other schizophrenia sub-groups. We propose that our results are of value to all patients with schizophrenia for several reasons. First, we demonstrated for the first time that there are three distinct behavioural similarities between TRS and treatment responsive patients. Specifically, TRS patients are less reward sensitive during effortful decision making when compared to controls [24–26], that this *insensitivity* is related to cognitive impairment [30], and that there is a divergence between the behavioural correlates of cognitive dysfunction and negative symptom severity [30]. Clearly, this demonstrates significant overlap in the behavioural deficits between treatment resistant and responsive patients, suggesting there may be common treatment targets, for example for interventions that improve sensitivity to reward. Where there has been a divergence has been in the relationship between negative symptom severity and effort sensitivity. While recent work has suggested negative symptom severity is associated with increased effort aversion, we show that in TRS it is associated with inefficient effort allocation. Could this be a TRS specific behavioural deficit? If so, this could represent a valuable behavioural divergence between TRS and treatment responsive patients which can be utilised for both diagnostic and treatment development purposes.

This question might be answered by additionally including a treatment responsive group for comparison which was lacking. This represents a limitation of this investigation which would be important to address in future work. Other limitations include a modest sample size, which while similar to other EBDM studies in schizophrenia, may limit the ability to further explore the relationships between cognitive sub-domains and decision making. Finally recent work in TRS has demonstrated that clozapine induced sedation may alter scores on negative symptom severity scales [64]. Specifically, improvements in motivation and pleasure may be underestimated due to medication induced sedation [64]. This may account for some of the differences in correlation between negative symptoms and behaviour in TRS and may need to be accounted for in future investigations.

To conclude, patients with TRS demonstrate significant behavioural deficits compared to controls when undergoing an EBDM task. Moreover, negative symptoms and cognitive dysfunction uniquely alter motivated behaviour in TRS, despite being positively associated with one another. This suggests that these factors contribute uniquely to motivated behaviour and may respond differentially to individually tailored therapy.

### Supplementary information

Supplementary Material

## Data Availability

Anonymised data and analysis code both available on request.

## Code availability

Anonymised data and analysis code both available on request.
