# Supplementary material for: Negative symptoms and cognitive impairment are associated with distinct motivational deficits in treatment resistant schizophrenia
Source: Mol Psychiatry. 2023 Aug 25;28(11):4831–41. doi: 10.1038/s41380-023-02232-7 (PMC10914595; doi:10.1038/s41380-023-02232-7)
Supplement: Supplementary file 1 — Supplementary Material [file 41380_2023_2232_MOESM1_ESM.docx]

**Negative symptoms and cognitive impairment are associated with**

**distinct motivational deficits in treatment resistant schizophrenia**

***Supplementary Material***

Literature review of EBDM studies

Supplementary Methods

Supplementary Results

# Literature review of EBDM studies

**Supplementary table 1: literature review EBDM studies in schizophrenia**

| Reference | Manuscript Citation number | Study design | Patient demographics | Mention of TRS/clozapine? |
| --- | --- | --- | --- | --- |
| Fervaha, 2013 | 24 | **Patients**: 16 patients with DSM-IV-TR diagnosis of Schizophrenia **Control group**: 16 healthy controls | - 18-35 years of age - Early in disease course - All patients stable on single atypical antipsychotic except one on dual therapy | No |
| Gold, 2013 | 26 | **Patients**: 44 with DSM-IV Dx of Schizophrenia or Schizoaffective disorder  **Control group**: 36 healthy controls | - Stable on medications for 4 weeks - 11 patients on 1^st^ generation, 19 on atypical, and 21 on clozapine. | Yes, however included in overall cohort without separating from treatment responsive patients. |
| Barch, 2014 | 25 | **Patients:** 59 patients with DSM-IV Dx of Schizophrenia or Schizoaffective disorder  **Control group**: 39 healthy controls | - 3 patients on typical, 46 on atypical, and 10 on combination. | No |
| Treadway, 2015 | 23 | **Patients:** 13 patients with DSM-IV Dx of Schizophrenia  **Control group**: 15 healthy controls | - All on antipsychotics, not specified for how long and if stable | No |
| Hartmann, 2015 | 29 | **Patients**: 31 patients with DSM-IV Dx of Schizophrenia or Schizoaffective disorder  **Control Group**: 20 healthy controls | - 28 on atypical antipsychotics and 3 on combination of typical + atypical | No |
| Reddy, 2015 | 27 | **Patients:** 94 patients with DSM-IV Dx of Schizophrenia  **Control Group:** 40 healthy controls | - 18-60 years of age - Clinically stable and no medication changes > 4 weeks - 77 on atypical, 11 on typical, 1 on both, 4 on no meds, 1 unclear medication status. | No |
| Moran, 2016 | 28 | **Patients:** 34 patients with DSM-IV Dx of Schizophrenia or Schizoaffective disorder  **Control Group:** No control group | - 32 on treatment (unspecified) 2 not on treatment. | No |
| McCarthy, 2016 | 61 | **Patients:** 48 patients with DSM-IV Dx of Schizophrenia or Schizoaffective disorder  **Control Group:** 27 healthy controls | - 18-60 years of age - All on medication but not specified | No |

# Supplementary Methods

**Ethics**

This study was approved by the local ethics committee (18/EE/0178; IRAS 237532). Informed written consent was obtained from all subjects and all testing was conducted in accordance with the declaration of Helsinki.

**Participants**

Inclusion criteria:

1. Aged between 18-65
2. Formally diagnosed with schizophrenia
3. Clinically stable on medication and an unchanged medication regime for at least 8 weeks

Exclusion criteria:

1. Participants who were unable to understand and engage with the behavioural testing protocol.
2. Physical disability preventing participants squeezing a hand-held device in response to visual stimuli.
3. Evidence of history suggestive of any concomitant neurodegenerative or neuroinflammatory disease such as Alzheimer’s disease or vascular dementia.

**Logistic regression with mixed effects**

This was completed in MATLAB using the *fitglme* function. Trials with reaction times less than 0.4s or greater than 10 seconds were deemed accidental and discarded. The first two models included a full factorial model exploring the effects of reward, effort and grouping on choice. Model two used a squared effort parameter. This variation was included as several studies have suggested that there is a quadratic relationship between force requirements and effort costs(4,5). A third model investigated the effect of cognitive function across all 80 participants on these groupwise differences. Finally a fourth model removed fixed factors (group, and cognitive function), and used an agnostic model that included per subject parameters of intercept, reward, effort and reward:effort. These parameters were used to predict behaviour, as well as in outcome variables in multiple regressions. The latter were conducted to associate behavioural measures with clinical variables within the schizophrenia patient group.

**Supplementary Table 2-1**. Model comparison of choice behaviour between schizophrenia and controls.

| Model | Model specification | Random effects | AIC** |
| --- | --- | --- | --- |
| 1 | 'Choice ~ Reward*Effort*Group’ | (Reward + Effort \| Subject) | 0 |
| 2 | 'Choice ~ Reward*Effort^2^*Group’ | (Reward + Effort^2^ \| Subject) | +2.6 |

** AIC reported in relation to the winning model, scaled at 0.

**Supplementary table 2-2. Effects of reward and effort on decision making.** Both controls and schizophrenia behave in a goal-directed manner, significantly altering responses based on reward levels, effort levels, and their interaction.

| **Group** | **Model** | **Effect of reward** | **Effect of effort** | **Reward by effort interaction** |
| --- | --- | --- | --- | --- |
| Control | Choice ~ reward*effort | F(1,4996) = 107, p < 0.0001 | F(1,4996) = 92.9, p < 0.0001 | F(1,4996) = 5.72, p = 0.01 |
| Scz | Choice ~ reward*effort | F(1,4981) = 61.5, p < 0.0001 | F(1,4981) = 57.3, p < 0.0001 | F(1,4981) = 4.1, p = 0.04 |

**Drift Diffusion Model**

Decisions to “accept” or “reject” offers on our effort-based decision making paradigm can be viewed as a two alternative forced choice task (2AFC) (6). Well validated modelling approaches such as drift diffusion modelling (DDM) can be used to analyse these types of data (6–9), and this approach has previously been used successfully to model choices with and without apathy in cerebrovascular small vessel disease (6). Here we used similar methods to investigate behavioural changes schizophrenia which are described below.

DDM frames the decision making process as a noisy accumulation of evidence towards one of two decision boundaries, beyond which a decision is made (i.e. accept or reject). The model can be encapsulated in four broad parameters: i) **bias**, *z*, which determines the *a priori* starting point of evidence accumulation in relation to a perfectly unbiased starting point (whose value = 0.5); ii) **threshold**, *a,*  which represents the distance between the two decision boundaries and the amount of evidence required to reach a decision; iii) **non decision time**, *t*, which accounts for biological processes not actively contributing to the decision making process (e.g. sensory perception, motor execution); iv) **drift rate**, *v*, which speaks to the rate of evidence accumulation.

As our task structure systematically varied reward and effort, we altered the drift rate in relation to the amount of reward and effort on offer. This gave rise to four sub-components of the parameter *v* per-subject:

i) baseline, or average, drift rate across all trials, *v_0_*

ii) effect of reward on drift rate, *v_r_*

iii) effect of effort on *v*, or *v_e_*

*iv)*  effect of the reward x effort interaction on *v,* or *v_r,e_*.

These components were used to compute an *actual drift rate*, *V_r,e_,* per subject within each segment of the decision making space. In line with previous work using this approach (6,7), we assumed this to be a linear combination of reward, effort and their interaction as portrayed below:

$$V_{r,e}=v_{0}+v_{r}+v_{e}+v_{r*e}$$

The noise parameter *c* was set to 1 by default. Population analysis was conducted with “subject” included as a hierarchical factor, followed by separate fits of each subject (6). Posterior distributions for *z, a, t, and V* (including its subcomponents) were generated. Between-group effects across DDM parameters were compared using Bayesian inference, and reported using posterior probabilities (P_P|D_). For Bayesian hypothesis testing, statistical significance was be inferred if the probability of a hypothesis being true was > 0.95. Within-group associations between DDM parameters and clinical measures in the schizophrenia group were conducted by averaging per-subject posterior distributions into a single point estimate. These were subsequently regressed against clinical measures of interest using robust multiple regressions. Statistical significance was set at p < 0.05.

Data was fit to a DDM using a well validated toolbox (10)(for further details: http://ski.clps.brown.edu/hddm_docs/; version 0.8.0; Python 3.6). Informative group mean priors used in the original toolbox were retained, as they constrain parameter estimates within a plausible range in accordance with previous literature (10,11). Samples from the joint posterior distributions for all parameters were generated using Markov chain Monte Carlo methods (MCMC). Ten thousand samples were performed per MCMC chain, with the first 1000 samples discarded as ‘burn-ins’. Five percent of the data was assumed to contain outliers and subsequently excluded. posterior predictive checks were conducted to ensure model predictions could accurately retrieve behavioural patterns in the original data set (**Fig. 5**).

**DDM model evaluation**

**Supplementary Table 3**: **Gelman Ruben statistic (R-hat) across five MCMC chains.** Appropriate model convergence in our model as demonstrated by R-hat values < 1.1 across all seven model parameters.

| *DDM Parameters* | *R-Hat M1 (SCZ)* | *R-Hat M2 (CTRL)* |
| --- | --- | --- |
| *Drift rate (V)* | ﻿0.9999 | ﻿1.0004 |
| *V:Reward* | ﻿1.0000 | ﻿1.0000 |
| *V:Effort* | ﻿1.0001 | ﻿1.0001 |
| *V:Reward*Effort* | ﻿1.0000 | ﻿1.0000 |
| *Threshold (a)* | ﻿1.0005 | ﻿1.0019 |
| *Non-decision time (t)* | ﻿1.0000 | ﻿1.0000 |
| *Bias (z)* | ﻿1.0006 | ﻿1.0006 |


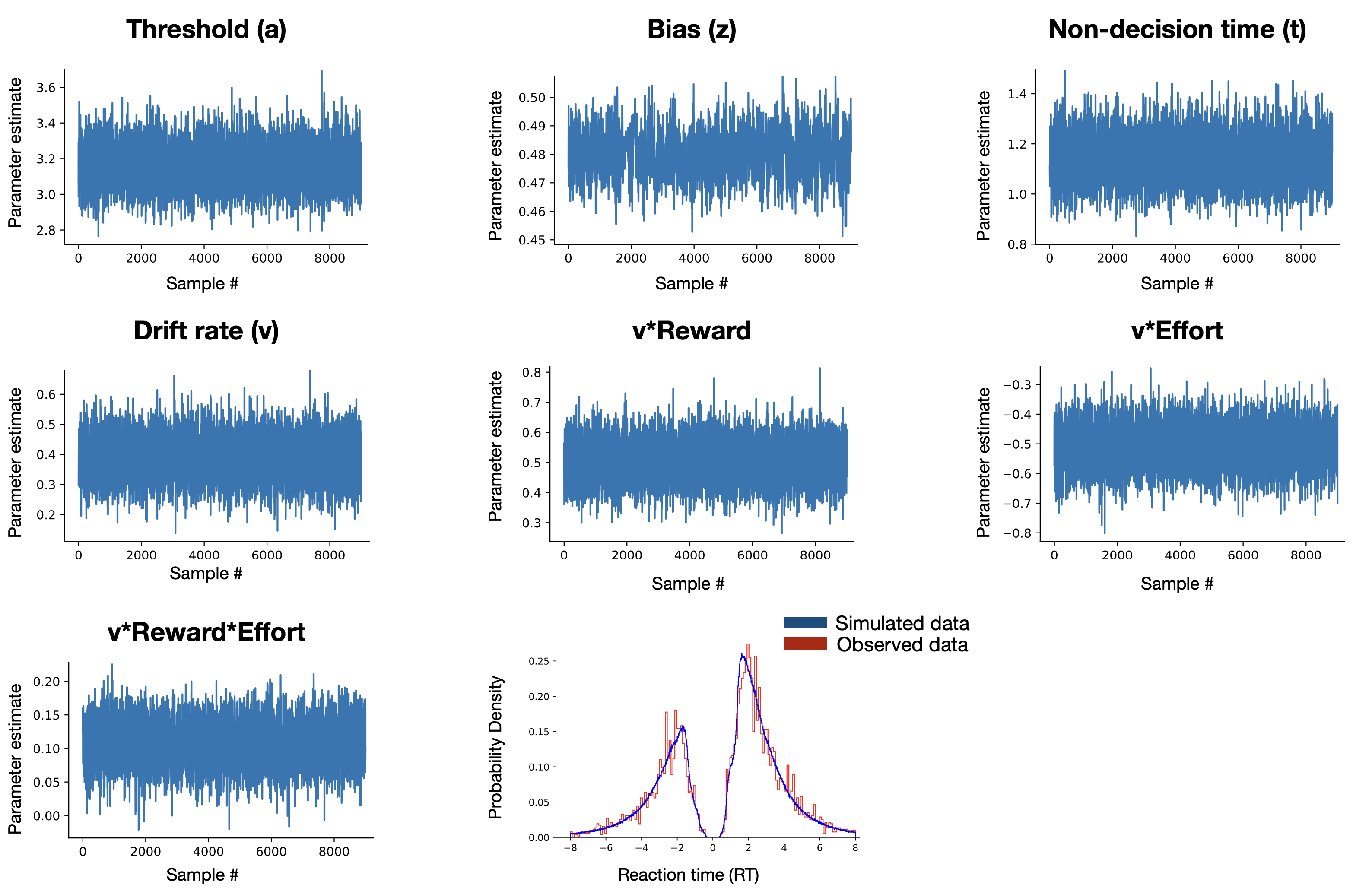


**Supplementary Figure 1**: **Model convergence and posterior predictive plots M1.** All seven model parameters appropriately converged after 10,000 Markov chain Monte Carlo samples. The first 1,000 samples have been discarded as burn-in and are not shown here. Posterior predictive plot for all patients**.** Probability density plot for all subjects’ raw reaction time (RT) data (Red) in comparison to our model’s predictions (blue). The two peaks represent the accepted trials’ RT (positive x-axis values) and rejected trials’ RT (negative values). Similar analysis can be seen below for M2.

**
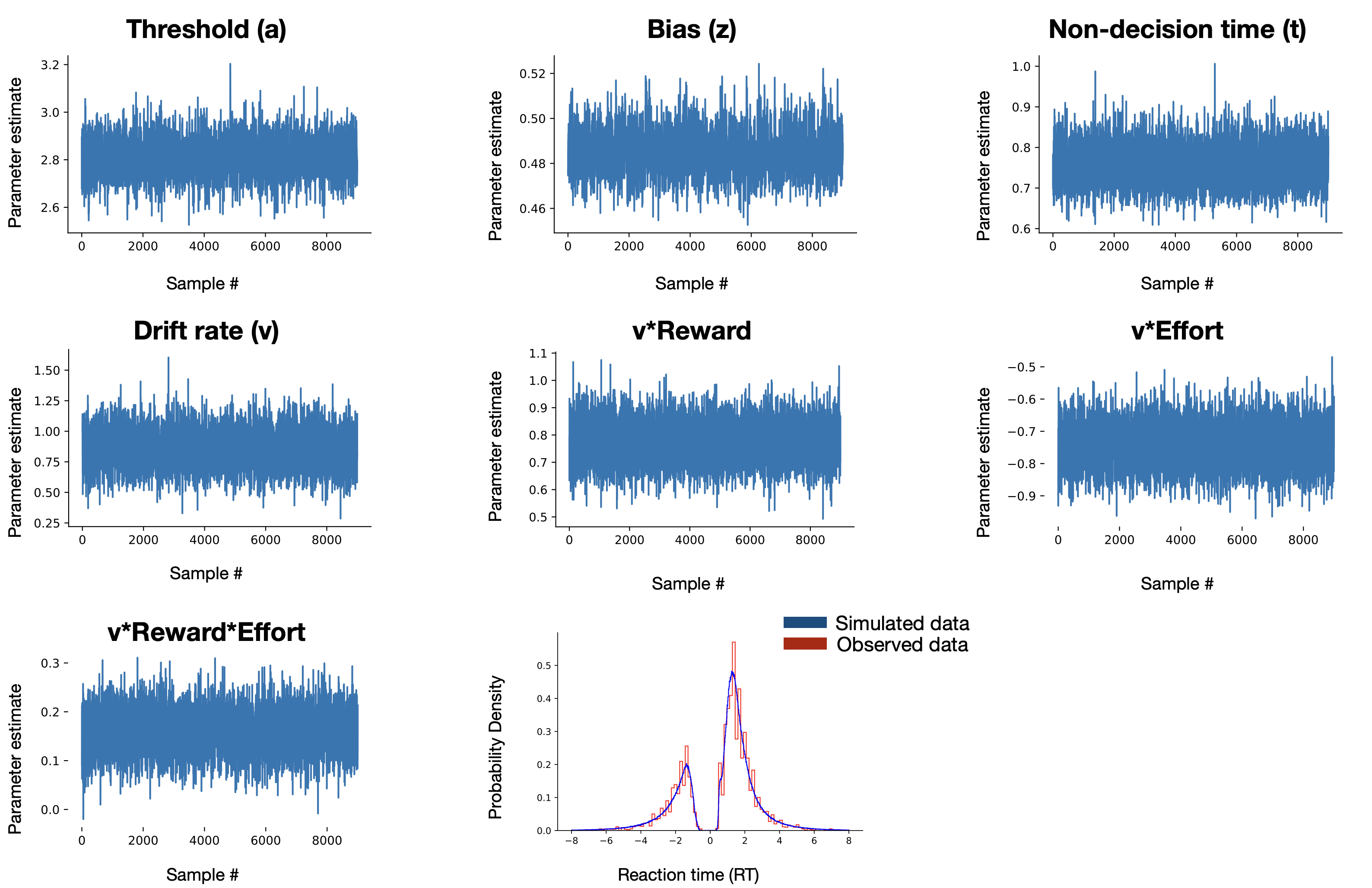
**

**Supplementary Figure 2**: **Model convergence and posterior predictive plots** **M2**

# Supplementary Results

**Questionnaire correlations:**

**Supplementary Figure 3. Clinical associations within schizophrenia patients.**

A visualisation of significant associations within a pairwise comparison using the corrplot function in R. Colour indicates the direction of associations so that blue and red represent positive and negative associations respectively. Only significant correlations (i.e. p < 0.05) are shown. Correlation strength, as measured by Pearson’s *r,* is represented by the width of the ellipses so that stronger correlations produce thinner the ellipses. Correlations are ordered along the first principle component to visually cluster associations. ACE_Total = Addenbrooke’s cognitive examination version III total score; PANS_Positive = Positive sub score of positive and negative syndrome scale for schizophrenia; BACS_Total = Brief assessment of cognition in schizophrenia scale; Cloz_level = serum clozapine level; CDS_Total = Calgary depression scale total score; STRAUSS_MAP = Motivation and pleasure factor of BNSS questionnaire using factor weightings from Strauss et al; STRAUSS_EXP = Blunted expression factor of BNSS questionnaire using factor weightings from Strauss et al.

# Cognitive variables:

Both ACE-III and BACS total scores were independently positively associated with reward sensitivity (respectively, [F(1,4852)=13.39, *P*=0.0002] and [F(1,4852)=4.481, *P*=0.035]). Including both scores in a logistic multiple regression with mixed effects retained the association between reward sensitivity and ACE-III but not BACS (respectively, [F(1,4352)=5.6, *P*=0.018] and [F(1,4352)=0.004, *P*=0.94]). Hence the ACE-III was deemed a more sensitive measure when correlating cognitive function and behavioural markers using our paradigm. Post-Hoc analyses can be seen below.

**Supplementary Table 4**: Association of ACE-III subdomains with reward sensitivity

| Subdomain | DF | F-statistic | uncorrected P value | Corrected p-value |
| --- | --- | --- | --- | --- |
| Attention | 1,4852 | 12.29 | 0.00046 | 0.0023** |
| Fluency | 1,4852 | 3.29 | 0.069 | 0.34 |
| Language | 1,4852 | 9.72 | 0.0018 | 0.009** |
| Visuo-Spatial | 1,4852 | 7.02 | 0.008 | 0.04* |
| Memory | 1,4852 | 6.76 | 0.0094 | 0.047* |

# Correlating behavioural parameters and clinical measures.

Below are multiple regressions of behavioural parameters extracted from the two analytic approaches. First the generalised models which used only the choice data (Supp. Table 4). Second the DDM models which combined choice data and reaction times (Supp. Table 6).

## Generalised linear mixed effects model parameters

**Supplementary Table 5**: Association between choice parameters and clinical measures

| DF(1,31) | **Intercept** | **Reward sensitivity** | **Effort sensitivity** | **Reward*Effort** |
| --- | --- | --- | --- | --- |
| Cognition (ACE-III) | β = 0.24  t = 0.83  p = 0.41 | β = 0.87  t = 3.32  p = 0.002  p(corr) = 0.014 | β = 0.03  t = 0.15  p = 0.88 | β = 0.25  t = 2.07  p = 0.046  p(corr) = 0.28 |
| Depression (CDS) | β = - 0.1  t = -0.33  p = 0.74 | β = -0.1  t = -0.36  p = 0.72 | β = 0.28  t = 1.00  p = 0.32 | β = -0.08  t = -0.64  p = 0.52 |
| Clozapine level | β = -0.44  t = -1.6  p = 0.11 | β = 0.16  t = 0.67  p = 0.51 | β = -0.60  t = -2.56  p = 0.015*  p(corr) = 0.09 | β = 0.014  t = 0.12  p = 0.90 |
| Positive Sx (PANS positive) | β = -0.27  t = -0.88  p = 0.38 | β = -0.21  t = -0.75  p = 0.46 | β = -0.096  t = -0.35  p = 0.72 | β = -0.092  t = -0.70  p = 0.49 |
| EXP | β = 0.1  t = 0.98  p = 0.33 | β = 0.077  t = 0.77  p = 0.45 | β = 0.28  t = 2.94  p = 0.006  p(corr) = 0.036 | β = 0.10  t = 2.20  p = 0.035  p(corr) = 0.21 |
| MAP | β = -0.14  t = -1.37  p = 0.18 | β = -0.06  t = -0.63  p = 0.53 | β = -0.18  t = -2.00  p = 0.054 | β = -0.04  t = -0.96  p = 0.34 |

## Drift Diffusion model parameters

**Supplementary Table 6**: Association drift diffusion model parameters and clinical measures

| DF(1,31) | Threshold (a) | Decision time (t) | Bias (z) | Drift rate | Drift * Reward | Drift *Effort | Drift*Rew*Eff |
| --- | --- | --- | --- | --- | --- | --- | --- |
| Cognition (ACE-III) | β = -0.076  t = -0.69  p = 0.49 | β = 0.16  t = 1.97  p = 0.058 | β = -0.00012  t = -0.14  p = 0.89 | β = 0.006  t = 0.12  p = 0.90 | β = 0.15  t = 3.25  p = 0.0027  p(corr) = 0.016 | β = 0.036  t = 0.72  p = 0.47 | β = 0.015  t = 0.87  p = 0.39 |
| Depression (CDS) | β = -0.05  t = -0.455  p = 0.65 | β = -0.15  t = -1.7  p = 0.098 | β = -0.00001  t = -0.013  p = 0.98 | β = -0.036  t = -0.58  p = 0.56 | β = 0.019  t = 0.38  p = 0.71 | β = 0.051  t = 0.94  p = 0.35 | β = -0.0020  t = -0.14  p = 0.89 |
| Clozapine level | β = -0.49  t = -0.48  p = 0.63 | β = -0.0022  t = -0.029  p = 0.97 | β = -0.0004  t = -0.49  p = 0.63 | β = -0.051  t = -0.97  p = 0.34 | β = -0.01  t = -0.23  p = 0.81 | β = -0.085  t = -1.83  p = 0.07 | β = -0.020  t = -0.123  p = 0.23 |
| Positive Sx (PANS positive) | β = -0.17  t = -1.4  p = 0.17 | β = 0.06  t = 0.74  p = 0.46 | β = -0.0007  t = 0.82  p = 0.41 | β = -0.05  t = -0.85  p = 0.40 | β = 0.02  t = 0.42  p = 0.68 | β = -0.042  t = -0.77  p = 0.44 | β = -0.0026  t = -0.14  p = 0.89 |
| EXP | β = -0.0066  t = -0.15  p = 0.88 | β = -0.039  t = -1.27  p = 0.21 | β = -0.0002  t = -0.58  p = 0.56 | β = 0.017  t = 0.82  p = 0.42 | β = 0.025  t = 1.43  p = 0.16 | β = 0.048  t = 2.58  p = 0.015  p(corr) = 0.09 | β = 0.0009  t = 1.48  p = 0.14 |
| MAP | β = -0.045  t = 1.13  p = 0.26 | β = 0.07  t = 2.44  p = 0.02  p(corr) = 0.12 | β = -0.0002  t = -0.67  p = 0.50 | β = -0.033  t = -1.6  p =0.11 | β = -0.023  t = -1.37  p = 0.18 | β = 0.13  t = -1.56  p = 0.12 | β = -0.0005  t = -0.81  p = 0.43 |
